# Mitochondrial genomic analyses provide new insights into the “missing” *atp8* and adaptive evolution of Mytilidae

**DOI:** 10.1186/s12864-022-08940-8

**Published:** 2022-11-02

**Authors:** Baojun Zhao, Shengtao Gao, Mingyang Zhao, Hongyu Lv, Jingyu Song, Hao Wang, Qifan Zeng, Jing Liu

**Affiliations:** 1 MOE Key Laboratory of Marine Genetics and Breeding, College of Marine Life Sciences, Ocean University of China, Qingdao, 266003 China; 2 Key Laboratory of Tropical Aquatic Germplasm of Hainan Province, Sanya Oceanog Inst, Ocean University of China, Sanya, 572000 China; 3 Laboratory for Marine Fisheries Science and Food Production Processes, Qingdao National Laboratory for Marine Science and Technology, Qingdao, 266237 China

**Keywords:** Mitochondrial genome, Mytilidae, *atp8*, Molecular phylogeny, Positive selection

## Abstract

**Background:**

Mytilidae, also known as marine mussels, are widely distributed in the oceans worldwide. Members of Mytilidae show a tremendous range of ecological adaptions, from the species distributed in freshwater to those that inhabit in deep-sea. Mitochondria play an important role in energy metabolism, which might contribute to the adaptation of Mytilidae to different environments. In addition, some bivalve species are thought to lack the mitochondrial protein-coding gene ATP synthase F0 subunit 8. Increasing studies indicated that the absence of *atp8* may be caused by annotation difficulties for *atp8* gene is characterized by highly divergent, variable length.

**Results:**

In this study, the complete mitochondrial genomes of three marine mussels (*Xenostrobus securis*, *Bathymodiolus puteoserpentis*, *Gigantidas vrijenhoeki*) were newly assembled, with the lengths of 14,972 bp, 20,482, and 17,786 bp, respectively. We annotated *atp8* in the sequences that we assembled and the sequences lacking *atp8*. The newly annotated *atp8* sequences all have one predicted transmembrane domain, a similar hydropathy profile, as well as the C-terminal region with positively charged amino acids. Furthermore, we reconstructed the phylogenetic trees and performed positive selection analysis. The results showed that the deep-sea bathymodiolines experienced more relaxed evolutionary constraints. And signatures of positive selection were detected in *nad4* of *Limnoperna fortunei*, which may contribute to the survival and/or thriving of this species in freshwater.

**Conclusions:**

Our analysis supported that *atp8* may not be missing in the Mytilidae. And our results provided evidence that the mitochondrial genes may contribute to the adaptation of Mytilidae to different environments.

**Supplementary Information:**

The online version contains supplementary material available at 10.1186/s12864-022-08940-8.

## Introduction

Mitochondria are essential eukaryotic organelles, they play important role in ATP (the universal currency of biological energy) production through oxidative phosphorylation (OXPHOS) [1]. The typical mitochondrial genome of animals is a small (16 kb) circular molecule, which includes 13 OXPHOS-related genes, 22 transfer RNA (tRNA) genes and 2 ribosomal RNA (rRNA) genes [1, 2], and it usually follows a strictly maternal inheritance. In bivalves, some species of Mytilidae [2, 3], Donacidae [4] and etc. showed a unique Doubly Uniparental Inheritance (DUI) model. In this model, there are two highly divergent male (M-type) and female (F-type) mitochondrial genomes (M-type vs F-type DNA divergence exceeds 20%) [1, 5]. Females with DUI possess only F-type, and males possess two types, but transmit only M-type to their sons. The mitochondrial genomes of bivalve species are also characterized by extraordinary variability in gene arrangement, tRNA gene number, and genome size. And some bivalve species are thought to lack the mitochondrial protein-coding gene ATP synthase F0 subunit 8 (ATP8) [6–8]. The presence and absence of *atp8* were mainly studied in Mytilidae, and *atp8* gene has been identified and proved to be actively transcribed and translated in *Mytilus spp.* [6, 9, 10]. However, the *atp8* gene of *Limnoperna fortunei* was presumed to be a pseudogene. Whether *atp8* gene was actually “missing” in some species has become a concern for researchers [5].

Mytilidae, also known as marine mussels, are widely distributed in the oceans worldwide. Some mussels are important economic species, for instance, *Mytilus chilensis*, *Mytilus. edulis, Mytilus coruscus, Perna viridis* [11, 12]*.* According to the Fishery and Aquaculture Statistics 2018 reported by Food and Agriculture Organization, the total production of *M. chilensis* (major species) in 2018 was 365,595 tonnes. Members of Mytilidae show a tremendous range of ecological adaptions, from the species distributed in freshwater to those that inhabit in deep-sea. The deep-sea environment is one of the most extreme environments on Earth, with limited food, low oxygen, high hydrostatic pressure, toxic chemicals and extreme temperature [13]. The species of Mytilidae that invaded deep-sea environments are mainly in the subfamily Bathymodiolinae. The evolutionary stepping stone hypothesis believes that the ancestors of Bathymodiolinae progressively adapted to deep-sea environments by exploiting sunken wood and whale carcasses [14]. Bathymodioline species usually have reduced digestive systems [15] and rely instead on endosymbiotic bacteria, transmitted horizontally from the environment to gill tissues, which produce organic carbon with energy from hydrogen sulfide oxidation. [16]. *L. fortunei*, golden mussel, is a species of Mytilidae with freshwater independent colonization [6, 17]. In freshwater, the low levels of ionic concentration may force organisms to expend more energy regulating osmotic pressure [18]. Given the functional importance of OXPHOS, mutations of the mitochondrial genes can directly affect metabolic performance. Mounting evidence suggests that some non-neutral mutations in mitochondrial genes can contribute to the adaptation of animals to different environments [19–21].

Mitochondrial DNA has been one of the most useful tools that are widely used in species identification, phylogenetic studies [22], comparative genomics [23], and management of invasive alien species [24]. *Xenostrobus securis, L. fortunei, and Mytilus galloprovincialis* and etc., are regarded as notorious invasive species which have caused dramatic and devastating effects on ecosystems [25, 26]. However, the complete mitochondrial genome of *X. securis* is still unknown. In addition, more mitochondrial genomes may contribute to further understanding the differentiation and evolution of Mytilidae [27, 28]. The emergence of cost-efficient next-generation sequencing allows us to quickly obtain mitochondrial genomes from various data (genomic data, transcriptome data, and metagenomic data) [29, 30]. In the present study, the complete mitochondrial genomes of *X. securis*, and two deep-sea mussels (*Bathymodiolus puteoserpentis*, *Gigantidas vrijenhoeki*) were newly assembled. We re-annotated *atp8* gene in Mytilidae, which is aim to answer whether *atp8* is not missing in the whole family. Furthermore, we also performed positive selection analysis of 12 protein-coding genes. We aim to provide new insights into the molecular mechanisms of adaptive evolution (to different environments: deep-sea and freshwater) of Mytilidae.

## Materials and methods

### Sequences and annotation

The sequencing data were download from NCBI (*X. secures* SRR7751554, *B. puteoserpentis* ERR3959529, *G. vrijenhoeki* SRR10802050) and filtered by Trimmomatic 0.36 [31–33]. The mitochondrial genomes of those species were assembled with the NOVOPlasty software [30]. The MITOS web server (http://mitos2.bioinf.uni-leipzig.de/index.py) was used to annotate the mitochondrial genomes [34]. tRNA genes were also predicated by ARWEN v1.2.3 (http://130.235.244.92/ARWEN/) [35]. The AT and GC skews were calculated according to the following formulae: AT-skew = (A − T)/(A + T) and GC-skew = (G − C)/(G + C).

Because of the small size and high variability of *atp8*, it is difficult for automatic annotation tools [5, 36]. The *atp8* sequences were annotated by manually scanning the intergenic regions. ORFfinder (https://www.ncbi.nlm.nih.gov/orffinder/) was used to find the ORFs. The start codon of *atp8* sequences was corrected according to the sequences of related species. TMHMM Server v.2.0 (http://www.cbs.dtu.dk/services/TMHMM/) was used to identify the transmembrane helices of *atp8* sequences. The PROTSCALE tool of ExPASy (http://ca.expasy.org/tools/) was applied to calculate the hydrophobicity profiles. In addition, we also annotated the *atp8* with HHblits v3.30 [37] referring to a previous study [38]. In brief, A Hidden Markov Model (HMM) was constructed for each ORF using HHblits with PDB70. An HMM for known *atp8* genes was constructed with the latest Uniclust30 database. Then, the HMM-HMM alignment was run against ORFs with *atp8*.

### Phylogenetic analyses

In this article, only F-type was included in the analyses. The 12 protein-coding genes of 46 sequences were used to reconstruct the phylogenetic relationships [39]. The *Crassostrea gigas* (AF177226.1) and *Atrina pectinata* (KC153059.1) served as outgroups (Table 1). *atp8* was excluded in the phylogenetic analysis as *atp8* was highly variable in length and amino acid composition. The sequences were aligned with Muscle in MEGA7 [40]. The gap and ambiguously aligned sites were recognized and removed with Gblocks Version 0.91b [41]. ModelTest-NG was used to identify the best-fit models for each gene based on the Akaike Information Criterion (AIC) [42]. Bayesian phylogenetic inference was performed with Mrbayes 3.2.7 [43]. Two independent Markov chain Monte Carlo (MCMC) simulations were carried out with four chains (one cold, three hot) for 1,000,000 generations, sampling every 1000 generations. The initial 25% of sampled trees were discarded as burn-in. Maximum Likelihood (ML) inference was performed using RAxML-NG with 1000 bootstrap replicates [44]. The phylogenetic trees were visualized by Figtree. v1.4.4.

**Table 1:** Complete mitochondrial genomes of Mytilidae used for phylogenetic analysis in this study

Species	Subfamily	Size (bp)	Accession number	Reference
*Limnoperna fortunei*	Limnoperninae	18,145	KP756905	[6]
*Lithophaga curta*	Lithophaginae	16,580	MK721546	[22]
*Bathmodiolus septemdierm*	Bathymodiolinae	17,069	AP014562	[45]
*Bathymodiolus marisindicus*	Bathymodiolinae	17,138	MT916745	[28]
*Bathymodiolus brooksi*	Bathymodiolinae	17,728	MT916743	[28]
*Bathymodiolus azoricus*	Bathymodiolinae	17,598	MT916742	[28]
*Bathymodiolus sp. 5 South*	Bathymodiolinae	18,376	MT916740	[28]
*Bathymodiolus puteoserpentis*	Bathymodiolinae	20,482	ON128252	This study
*“Bathymodiolus” thermophilus*	Bathymodiolinae	18,819	MK721544	[22]
*“Bathymodiolus” manusensis*	Bathymodiolinae	partial	KY270856	-
*Bathymodiolus aduloides*	Bathymodiolinae	17,243	MT916741	[28]
*Gigantidas japonicus*	Bathymodiolinae	17,510	AP014560	[45]
*Gigantidas securiformis*	Bathymodiolinae	17,199	KY270857	-
*Gigantidas platifrons*	Bathymodiolinae	17,653	AP014561	[45]
*Gigantidas childressi*	Bathymodiolinae	17,637	MT916744	[28]
*Gigantidas haimaensis*	Bathymodiolinae	18,283	MT916746	[28]
*Gigantidas vrijenhoeki*	Bathymodiolinae	17,786	ON128253	This study
*Modiolus modiolus*	Modiolinae	15,816	KX821782	[46]
*Modiolus kurilensis*	Modiolinae	16.210	KY242717	-
*Modiolus nipponicus*	Modiolinae	15,638	MK721547	[22]
*Modiolus comptus*	Modiolinae	15,591	MN602036	[47]
*Modiolus philippinarum*	Modiolinae	16,389	KY705073	-
*Xenostrobus securis*	Arcuatulinae	14,972	ON128254	This study
*Septifer bilocularis*	Septiferinae	16,253	MK721549	[22]
*Perna viridis*	Mytilinae	16,014	JQ970425	[48]
*Perna canaliculus*	Mytilinae	16,005	MG766134	[49]
*Arcuatula senhousia*	Arcuatulinae	21,557	GU001953	[2]
*Gregariella coralliophaga*	Crenellinae	16,273	MK721545	[22]
*Mytilus chilensis*	Mytilinae	16,765	KP100300	[50]
*Mytilus edulis*	Mytilinae	16,745	MF407676	[10]
*Mytilus galloprovincialis*	Mytilinae	16,780	FJ890849	[51]
*Mytilus trossulus*	Mytilinae	18,628	HM462080	[52]
*Mytilus californianus*	Mytilinae	16,730	GQ527172	[53]
*Crenomytilus grayanus*	Mytilinae	17,582	MK721543	[22]
*Mytilus coruscus*	Mytilinae	16,642	KJ577549	[54]
*Geukensia demissa*	Brachidontinae	15,838	MN449487	[55]
*Brachidontes mutabilis*	Brachidontinae	16,531	MK721541	[22]
Mytilaster solisianus*	Brachidontinae	18,415	KM655841	[56]
*Brachidontes exustus*	Brachidontinae	16,600	KM233636	[57]
*Perumytilus purpuratus S*	Brachidontinae	16,986	MH330333	[3]
*Perumytilus purpuratus N*	Brachidontinae	16,963	MH330332	[3]
*Semimytilus algosus*	Brachidontinae	18,113	MT026712	[58]
*Mytilisepta keenae*	Brachidontinae	15,902	MK721542	[22]
*Mytilisepta virgate*	Brachidontinae	14,703	MK721548	[22]

^*^The sequence (KM655841.1) may from *Mytilaster solisianus* rather than “*Perna Perna”*

The divergence time was estimated using the program MCMCtree in PAML4.9 [59]. Two nodes were used as calibrations, one of which was from the fossil recode data of Modiolinae (393–408 Mya) and the other was from previous studies [28, 60, 61], the time of divergence between *B. themophilus* and *G. childressi* was approximately 21.1–33.0 Mya.

### Selection analyses

Comparing the nonsynonymous/synonymous nucleotide substitution ratios (*ω* = *d*_*N*_/*d*_*S*_) has been widely used to evaluate the adaptive molecular evolution of protein-coding genes. The values of *d*_*N*_/*d*_*S*_ mean changes in selective pressure, where the *d*_*N*_/*d*_*S*_ < 1, = 1, > 1 correspond to negative purifying selection, neutral evolution and positive selection, respectively. The program CODEML in PAML4.9 was applied to calculate the values of *d*_*N*_/*d*_*S*_ [59]. The phylogenetic tree of 12 protein-coding genes inferred with Mrbayes was used for selection analyses. The outgroups were not included in selection analyses. For branch model, One-ratio model (model = 0, NSsites = 0, icode = 4) and Three-ratios model (model = 2, NSsites = 0, icode = 4) were performed. The deep-sea branches (*Bathymodiolinae*) and freshwater branches (*L. fortunei*) were used as foreground branches (two foreground branches) and the remaining were used as background branches. In addition, the branch-site model (model = 2, NSsites = 2) was used to determine whether positive selection acted on specific sites on foreground branches. The sites under positive selection were identified with Bayes empirical Bayes posterior probabilities (> 0.95). The likelihood ratio tests were carried out to identify if the alternative model provided a significantly better fit than the null model.

To explore the possible effects of positive selection sites on protein function, the three-dimensional structure of protein was predicted with phyre2 [62]. The protein structure of NuoM in *Escherichia coli* [63]was used as a template [21, 64]. The positive sites were marked using PyMOL.

## Results and discussion

### General features

We have successfully obtained the complete mitochondrial genomes of *X securis, B. puteoserpentis,* and *G. vrijenhoeki*, with lengths of 14,972 bp, 20,482, and 17,786 bp, respectively. The genomes we assembled showed high similarity with the known sequences of each species (100% for *X. securis*; 100% for *B. puteoserpents*; 99.42% for *G. vrijenhoeki*). It should be pointed that *X. secures* might be a cryptic species complex, and we cannot rule out the possibility that the mitochondrial genome of *X. securis* may belong to the M-type [65, 66]. The base composition analysis showed that three assembled genomes were biased toward A and T, with AT content of 59.08% in *X securis*, 63.55% in *B. puteoserpentis*, and 66.96% in *G. vrijenhoeki.* The assembled genomes are all characterized by negative AT skew and positive GC skew (Table 2). The base composition and skewness are consistent with most studies in bivalves [8, 67, 68].

**Table 2:** AT content, GC content, and compositional asymmetry of three mitogenomes

Species	AT%	GC%	AT skew	GC skew
*Xenostrobus securis*	59.08	40.92	-0.225	0.231
*Bathymodiolus puteoserpentis*	63.55	36.45	-0.247	0.270
*Gigantidas vrijenhoeki*	66.96	33.04	-0.225	0.294

**Fig. 1:**
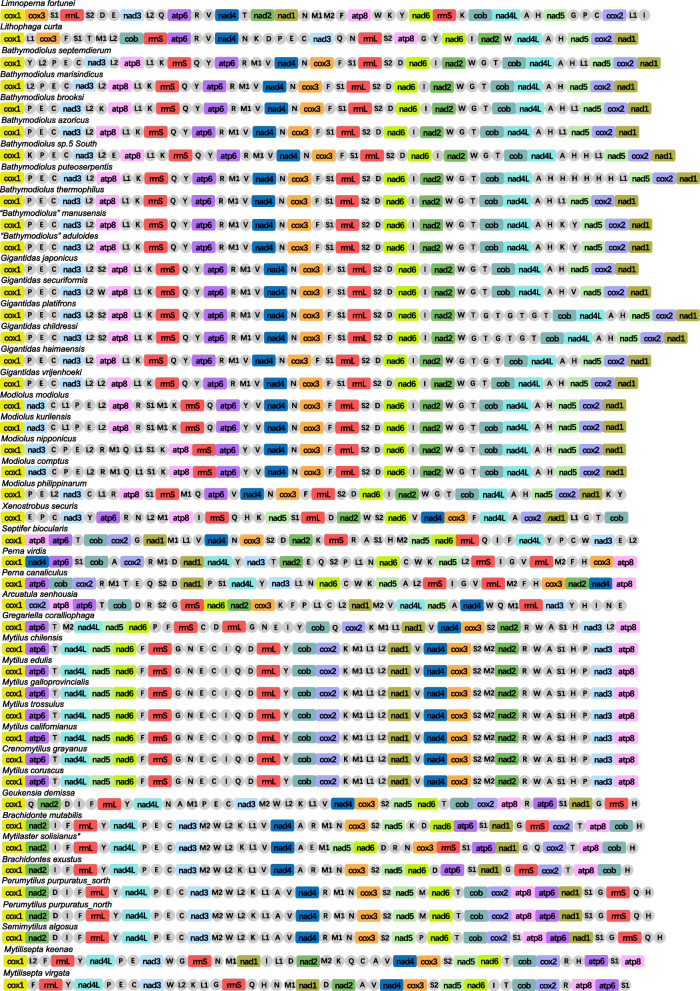
Linearized mitochondrial gene arrangement patterns of 44 Mytilidae sequences. Genome and gene size are not in scale. * Note: The sequence (KM655841.1) may from *Mytilaster solisianus* rather than “*Perna Perna”*

For these three species, all genes encoded on the heavy strand (H-strand) except tRNA Gly in Light (L-strand). Each genome has 13 protein-coding genes and 2 ribosomal RNA genes (Fig. 1). However, the number of tRNAs is varied. Twenty-two typical tRNAs were identified in *X securis*. 27 tRNAs (four more tRNA^His^ and one more tRNA^Leu^) and 23 tRNAs (one more tRNA^Leu^) were identified in *B. puteoserpentis* and *G. vrijenhoeki*, respectively. The lengths of intergenic region between tRNA^His^ were 470 bp, 441 bp, 455 bp and 468 bp, respectively, which leads *B. puteoserpentis* to have the largest mitochondrial genome among Bathymodiolinae. In the assembled genomes of *X securis*, *B. puteoserpentis,* and *G. vrijenhoeki,* the total lengths of protein-coding genes were 11,060, 10,947, and 10,993, accounting for 73.87%, 53.45%, 61.81% of the whole genome, respectively. The protein-coding genes of *X securis* started with ATG and ATA, while both of *B. puteoserpentis* and *G. vrijenhoeki* started with ATG, ATA, ATT, and GTG. For these three species, the protein-coding genes mainly started with codon ATG. The stop codons of all species were either TAA or TAG except *nad1* and *cox3* of *X. securis* which had an incomplete stop codon of T. The presence of incomplete stop codons is a common feature of the mitochondrial genes among animals [5, 69, 70]. The incomplete stop codon is thought to be completed by polyadenylation of the transcript.

### ATP8 annotation

**Fig. 2:**
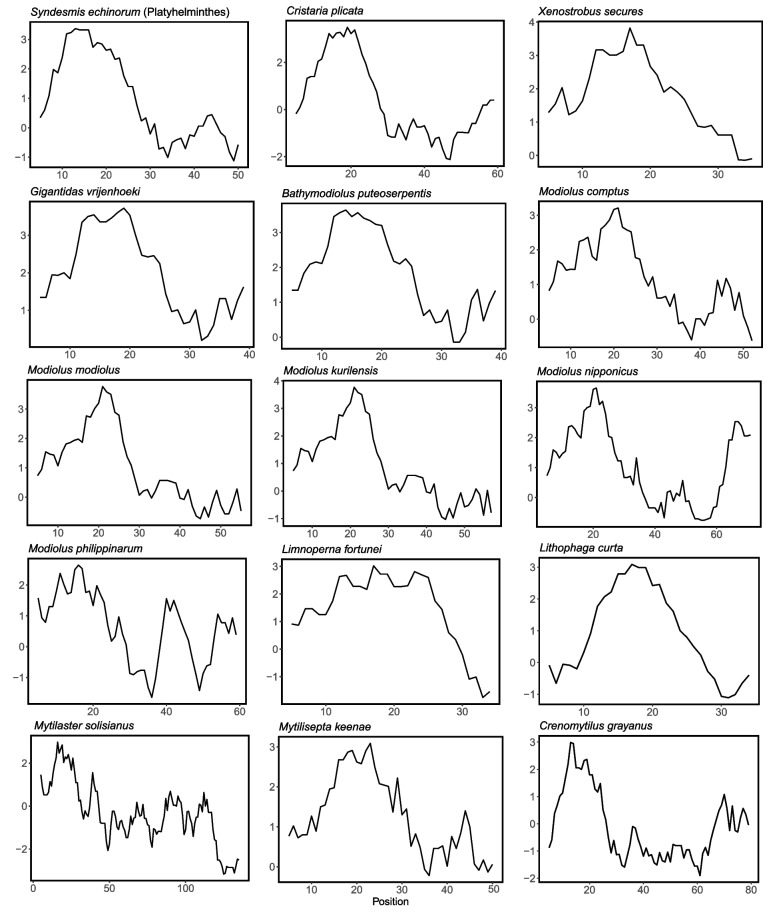
Hydropathy profile of candidate *atp8* gene identified in this study, in comparison with the previously inferred *atp8* gene (*Syndesmis echinorum*, MT063058; *Cristaria plicata*, KM233451)

**Fig. 3:**
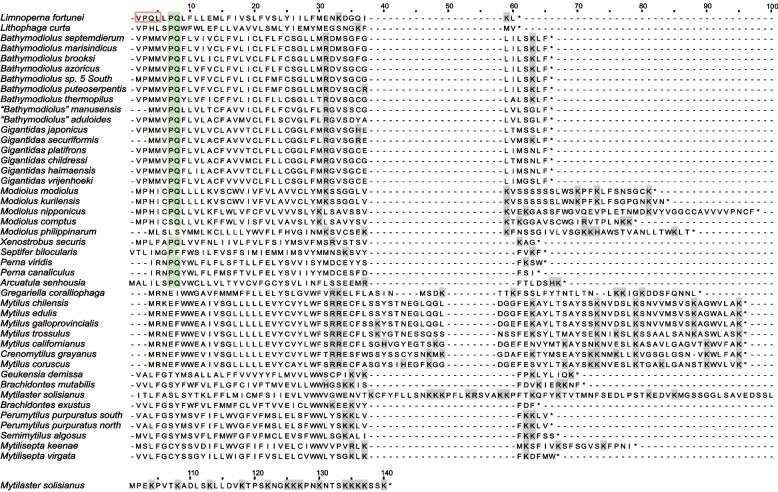
Alignment of *atp8* gene. The first column shows the species name. Red border: “MPQL” amino acid signature of *Limnoperna fortunei*; Green box: the “PQ” amino acid signature; Grey box: positively-charged amino acids

Some species are thought to lack *atp*8 gene that encodes a subunit of mitochondrial ATP synthase [6, 7]. Increasing studies indicated that the absence of *atp8* may be caused by annotation difficulties for *atp8* gene is characterized by highly divergent, variable length. Sometimes, *atp8* gene could not be detected by automatic annotation software, the annotation of *atp*8 gene usually requires manual inspection and comparison to *atp*8 sequences from other species. In this study, we manually annotated *atp8* in the sequences that we assembled and the sequences lacking *atp8*. Twelve *atp*8 sequences were manually annotated in the intergenic region (Table 3). The results of manual annotation were highly consistent with the results of HMM. However, HMM method was unable to detect *atp8* in some species (e.g. *L. fortunei*, *X. secures* and Modiolinae,), probably due to the lack of *atp8* sequences from related species and the low sequence similarity with known *atp8* genes. For newly annotated *atp*8, start codons were ATG or GTG or ATC, and stop codons were either TAG or TAA. ATP8 usually has higher conservation of the secondary structure compared to the primary sequence [71]. The newly annotated *atp*8 sequences all have one predicted transmembrane domain, a similar hydropathy profile, as well as the C-terminal region with positively charged amino acids (R, H, and K). (Table 3, Figs. 2 and 3) [72].

**Table 3:** Annotation of *atp8* gene in Mytilidae

Species	Position (bp)	Size (bp)	Intergenic region (bp)^a^	Start codon	Stop codon	TM^b^	GenBank	Annotation Methods^c^
*Modiolus modiolus*	3240–3419	180	194	ATG	TAA	7–29	KX821782	M
*Modiolus kurilensis*	676–861	186	191	ATG	TAA	7–29	KY242717	M
*Modiolus nipponicus*	3409–3636	228	304	ATG	TAA	13–35	MK721547	M
*Modiolus comptus*	3106–3276	171	670	ATG	TAG	13–35	MN602036	M
*Modiolus philippinarum*	16,113–16,304	192	209	ATG	TAA	4–26	KY705073	M
*Xenostrobus securis*	3119–3238	120	120	ATG	TAG	7–29	ON128254	M
*Limnoperna fortunei*	157–273	117	271	ATA	TAA	10–32	KP756905	M
*Mytilisepta keenae*	15,718–15,882	165	186	ATG	TAA	10–32	MK721542	M & HMM
*Lithophaga curta*	10,543–10,659	117	899	GTG	TAA	7–29	MK721546	M & HMM
*Bathymodiolus puteoserpentis*	3193–3324	132	241	GTG	TAG	7–29	ON128252	M & HMM
*Gigantidas vrijenhoeki*	3304–3435	132	157	GTG	TAA	7–29	ON128253	M & HMM
*Crenomytilus grayanus*	17,320–17,571	251	279	ATG	TAA	5–27	MK721543	M &HMM
*Mytilaster solisianus*	13,905–14,324	420	574	ATC	TAA	13–32	KM655841	M & HMM

^a^ Intergenic region used for annotation of *atp8*

^b^ *TM* Transmembrane

^c^ Annotation methods. *M* Manual annotation, *HMM* hhblits annotation

In this study, all species of Mytilidae possessed an annotated *atp8* gene, which allows us to further understand the features of *atp8* gene in a family. The lengths of *atp*8 in Mytilidae were short and variable, ranging from 37 – 139 aa (Table 3 and Fig. 3). The longest *atp8* was from *Mytilaster solisianus* (KM655841.1), and the shortest *atp8* was from *P. canaliculus*. It should be noted that the annotation of the start codons and stop codons might be inaccurate in some species due to the lack of additional data. The *atp8* sequence of *M. solisianus* was much longer than that of related species. We are not sure whether this sequence used an incomplete stop codon (TA or T), which caused the fact that the real length was shorter than the current length. The alignment of *atp8* gene indicated that *atp8* sequences were highly divergent that they showed similarity only in related species. The conserved ‘MPQL’ amino acid signature at the N-terminus, the typical characteristic for metazoan ATP8 proteins [71], was only found in *L. fortunei* (VPQL) (Fig. 3). However, the conserved ‘PQ’ amino acid signature was found in many species, for instance, Bathymodiolinae, Limnoperninae, Lithophaginae, *P. viridis*, *P. canaliculus*, *Arcuatula senhousia* and some species of Modiolinae [72]. Although not all species of Mytilidae have this feature, it still can contribute to identifying *atp*8 gene from ORFs in some species of Mytilidae.

Given the characteristics of *atp8* gene, it is not surprising that *atp8* gene was once presumed to have lost in many species. Although *atp*8 gene of *L. fortunei* has the ‘MPQL’ amino acid signature at the N-terminus, it was still annotated as a pseudogene in an incorrect position [6]. In almost all lineages of animals, there has been strong selection to maintain a minimal set of 37 genes [5]. Researchers need to be cautious of assertions that a mitochondrial gene is missing [73]. Our results supported that *atp*8 gene may not be missing in the Mytilidae. Although we have no right to claim that whole Bivalvia class possesses an *atp*8 gene, we provided further evidence that a family possesses the *atp*8 gene. In the future, studies of transcriptional activity and function of these *atp*8 genes may be necessary. Moreover, we strongly encourage researchers to identify whether *atp*8 gene was not missing in other families.

### Phylogenetic relationship within Mytilidae

**Fig. 4:**
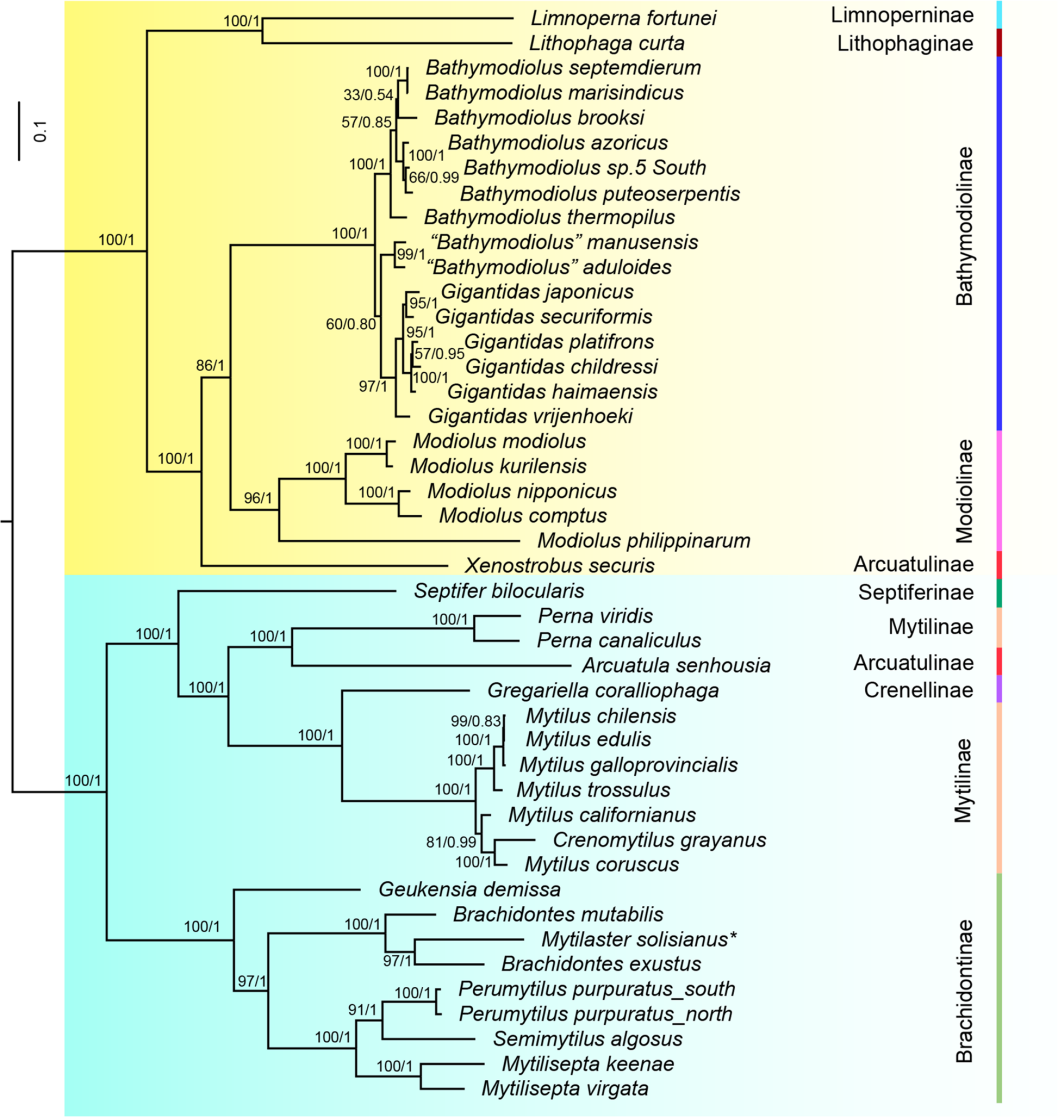
Phylogenetic relationships of Mytilidae species based on 12 protein-coding genes using Bayesian inference and maximum likelihood methods. * Note: The sequence (KM655841.1) may from *Mytilaster solisianus* rather than “*Perna Perna”*

**Fig. 5:**
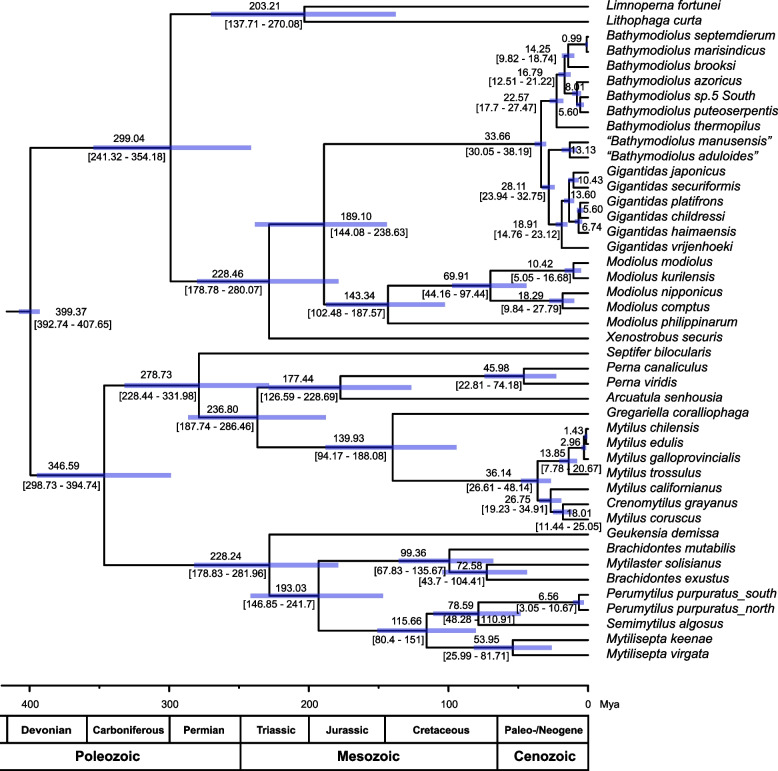
Divergence time estimation of Mytilidae inferred with MCMCtree in Paml. Shaded bars on nodes indicate 95% highest posterior density (PHD) intervals for each node

To further examine the relationship among the Mytilidae species, the phylogenetic trees were reconstructed using Maximum Likelihood and Bayesian inference methods with a concatenated alignment. The tree topologies resulting from these two methods were consistent. The results supported that the Mytilidae is subdivided into two major clades [22]. The clade 1 contained the subfamilies Bathymodiolinae, Modiolinae, Limnoperninae, and Lithophaginae and the genus *Xenostrobus* (Arcuatulinae), and clade 2 included subfamilies Brachidontinae, Mytilinae, Crenellinae, Septiferinae, and genus *Arcuatula* (Arcuatulinae) (Fig. 4). The estimated divergence time between the two clades was around 399.37 Mya (95% HPD interval 392.74- 407.65 Mya), which is close to the estimated time in other analyses (Fig. 5) [22, 74].

The subfamily Bathymodiolinae was monophyletic, which is the same with previous studies [28, 60]. In this study, the Bathymodiolinae were divided into three separate clades, corresponding to the *Gigantidas*, *Bathymodiolus,* and *“Bathymodiolus”*. The Gigantidas was clustered with *“Bathymodiolus”* and then sister to *Bathymodiolus*, which is consistent with previous analysis [60], but different from zhang’s study [28]. It should be noted that although the Gigantidas clustered with *“Bathymodiolus”*, the node was not supported enough according to bootstrap value and posterior probability. Our results indicated that the subfamily Arcuatulinae was polyphyletic as genera *Xenostrobus* and *Arcuatula* were divided into the clade1 and clade2, respectively. In clade1, the genus *Xenostrobus* and (Modiolinae + Bathymodiolinae) were grouped in a subclade with high supporting values (100% BP and 1.00 BPP). The placement of Genus *Xenostrobus* was different between our results and a previous study based on 5 genes [74]. The tree of the previous study showed that *Xenostrobus* was clustered with Bathymodiolinae and then sister to Modiolinae. However, the gene order of 13 protein-coding genes and 2 rRNA (excepting tRNA) between Modiolinae and Bathymodiolinae was consistent, which supported our result (Fig. 1). Further increasing the sequences of *Xenostrobus* may contribute to resolving the phylogenetic relationship among Genus *Xenostrobus*, Modiolinae, and Bathymodiolinae.

In clade 2, Brachidontinae were divided into three well-supported clades: [1] *Geukensia* [2] *Brachidontes* [3] *Mytilisepta* + *Perumytilus* + *Semimytilus*, which was similar to the results of nuclear genes18S and 28S [75]. However, the placement of *Geukensia* was inconsistent. Moreover, a previous study [22] and our result indicated that *Perna perna* (KM655841.1) had an unusual phylogenetic status, which showed high similarity with two *Brachiodontes* species rather than *P. viridis* and *Perna canaliculus* according to gene order and phylogenetic trees (Figs. 1 and 4) [22]. The sequence of *P. perna* (KM655841.1) showed 99.83% sequence identity with *cox1* sequences of *M. solisianus*, which suggested that the sequence may belong to *M. solisianus* rather than *P. perna*.

### Positive selection analyses

Purifying selection has been widely recognized as the predominant force acting on the molecular evolution of mitochondrial genomes. However, some studies have demonstrated that relaxation of purifying selection or episodic positive selection on mitochondrial genomes may occur in species that have different types of locomotion [76] or species living in extreme environments [77–79]. The One-ratio model analysis the *ω* values of these 12 genes ranged from 0.0024 to 0.0435, where *cox1-3* have lower *ω* values than other genes (Table 4). All the *ω* values were less than 1, indicating that the 12 genes of Mytilidae experienced constrained selection pressure to maintain their function. Members of Mytilidae show a tremendous range of ecological adaptions. To examine whether heterogeneous selective pressures act on the branches living in different environments (freshwater, deep-sea, and shallow sea), the Three-ratios model analysis was implemented. The likelihood ratio tests showed that the Three-ratios models have significantly better fit than the null models at *cox1*, *atp6*, *cob*, *nad2,* and *nad5* (Table 4), suggesting divergence in selective pressure among the branches. In deep-sea branches, the *ω* values of those genes excepting *cox1* are higher than those of other branches, suggesting those genes experienced relaxation of purifying selection. Relaxation of purifying selection in deep-sea branches has been found in many studies including deep-sea sea cucumbers and *Boudemos sp.* (Calamyzinae) [77, 80]. The relaxed purifying selection may be beneficial for deep-sea species to adapt to the reduction of oxygen levels and metabolic rates in extreme environments. In freshwater branches, only the ω value of *atp6* was higher than that of shallow-sea branches, but still lower than the ω value of deep-sea branches.

**Table 4:** Branch model analyses in Mytilidae

Genes	One-ratio (lnL)	ω	Three-ratios (lnL)	Shallow sea (ω)	Deep-sea (ω)	Freshwater (ω)	*p*-value
*atp6*	-9059.97	0.0147	-9048.60	0.0051	0.0227	0.1179	0.000
*cox1*	-25,065.71	0.0047	-25,058.99	0.0061	0.0024	0.0050	0.001
*cox2*	-8952.85	0.0024	-8952.57	0.0025	0.0019	0.0175	0.756
*cox3*	-15,865.81	0.0131	-15,863.72	0.0163	0.0102	0.0031	0.124
*cob*	-21,708.21	0.0207	-21,677.83	0.0109	0.0344	0.0028	0.000
*nad1*	-15,212.85	0.0244	-15,212.24	0.0236	0.0262	0.0107	0.543
*nad2*	-17,841.44	0.0433	-17,831.67	0.0336	0.0591	0.0048	0.000
*nad3*	-5483.56	0.0225	-5482.37	0.0186	0.0284	0.0025	0.304
*nad4*	-21,382.34	0.0370	-21,381.96	0.0366	0.0376	0.0024	0.684
*nad4L*	-4847.06	0.0312	-4846.12	0.0283	0.0371	0.0035	0.391
*nad5*	-35,424.55	0.0435	-35,414.81	0.0366	0.0528	0.0028	0.000
*nad6*	-6775.67	0.0255	-6772.78	0.0213	0.0333	0.0034	0.056

**Fig. 6:**
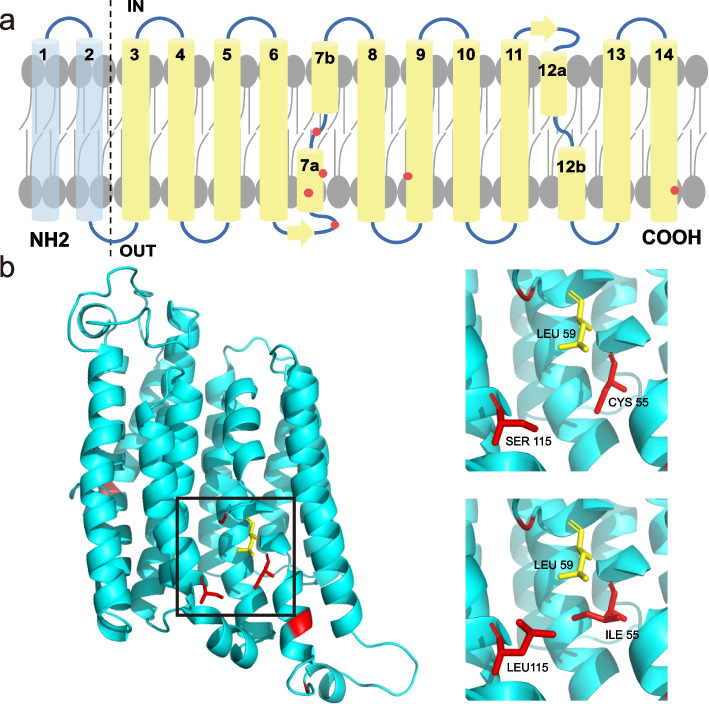
The structure analysis of *nad4* (**a**) The topology diagram of *nad4* of *Limnoperna fortunei*. In transparent blue, representation of N-terminal part not similar with *Escherichia Coli.* The positions of positive sites were indicated in red. **b** The structure analysis of positive sites. Upper-right side: *L. fortunei model*; Lower-right side: *Mytilus edulis* model. The positions of positive sites were indicated in red, and the amino acid in close proximity was indicated in yellow

To identify whether positive selection acts on a few sites in freshwater branches or deep-sea branches, the branch-site model analysis was carried out. In deep-sea branches, although several sites of the genes (*atp6*, *cob*, *nad2*, *nad4*, *nad5,* and *nad6*) were recognized as positive sites according to BEB analysis (> 95%), the p-values of likelihood ratio tests were > 0.05 (Table S1). In freshwater branches, sites of *nad2*, *nad4,* and *nad5* were identified as positive sites with BEB analysis (> 95%), however, only the p-value of *nad4* was significant, which means *nad4* may contribute to the adaptation of *L. fortunei* in freshwater (Table 5). Successful adaption to the freshwater environment may have required increased demand for energy involved in processes such as the osmotic balance [21]. NADH dehydrogenase, the largest and the most complicated enzyme of the respiratory chain, receives electrons from the oxidation of NADH and provides electrons for reduction of quinone to quinol [81]. *nad4* together with *nad2* and *nad5* were considered to be the actual proton pumping devices as they showed homology with a class of Na + / H + antiporters [82]. Mutation in the members of NADH dehydrogenase would change the metabolic capacity which may further affect the fitness of an organism. To explore the possible effects of positive selection sites on *nad4*, the protein model was generated using the *E. coli* structure as a template. Most of the positive sites were directly located in the TMα7a which plays the most important role in the transportation of hydrogen ion (Fig. 6a). A positive site was found near the end of TMα9, which is adjacent to a positive site located in TMα7a. Intriguingly, both positive sites are polar amino acids, and these substitutions could change the environment between TMα7a and TMα9 (Fig. 6b) [21, 83]. This possible interaction was similar to a previous study of *nad2* in freshwater dolphins [21]. We speculated that the mutations in NADH dehydrogenase may contribute to the survival and/or thriving of these species in freshwater.

**Table 5:** Branch-site model analyses in freshwater branches

Genes	Model	lnL	2△lnL	Parameter estimates	Positive sites	*P*-value
*nad2*	Alternative	-17,791.53	0	*P*_0_ = 0.648 *P*_1_ = 0.009 *P*_2a_ = 0.339 *P*_2b_ = 0.004 ω_0_ = 0.042 ω_1_ = 1.000 ω_2_ = 1.000	4S(0.954) 74 N(0.997) 110G(0.989)	1
	Null	-17,791.53		*P*_0_ = 0.648 *P*_1_ = 0.009 *P*_2a_ = 0.339 *P*_2b_ = 0.004 ω_0_ = 0.042 ω_1_ = 1.000 ω_2_ = 1.000		
*nad4*	Alternative	-21,314.63	9.38	*P*_0_ = 0.799 *P*_1_ = 0.030 *P*_2a_ = 0.165 *P*_2b_ = 0.006 ω_0_ = 0.039 ω_1_ = 1.000 ω_2_ = 209.31	41Y(0.956) 49S(0.979) 55L(0.975) 63 V(0.963) 115L(0.966) 281F(0.974)	0.002
	Null	-21,319.32		*P*_0_ = 0.796 *P*_1_ = 0.037 *P*_2a_ = 0.159 *P*_2b_ = 0.007 ω_0_ = 0.039 ω_1_ = 1.000 ω_2_ = 1.000		
*nad5*	Alternative	-35,070.30	3.10	*P*_0_ = 0.806 *P*_1_ = 0.074 *P*_2a_ = 0.110 *P*_2b_ = 0.010 ω_0_ = 0.044 ω_1_ = 1.000 ω_2_ = 13.12	346 N(0.982) 462Q(0.982)	0.078
	Null	-35,071.85		*P*_0_ = 0.793 *P*_1_ = 0.073 *P*_2a_ = 0.123 *P*_2b_ = 0.011 ω_0_ = 0.044 ω_1_ = 1.000 ω_2_ = 1.000

## Conclusions

Here, the mitochondrial genomes of three marine mussels (*Xenostrobus securis*, *Bathymodiolus puteoserpentis*, and *Gigantidas vrijenhoeki*) were assembled using the sequences deposited in NCBI. We annotated *atp8* in the sequences that we assembled and the sequences lacking *atp8*. The newly annotated *atp8* sequences all have one predicted transmembrane domain, a similar hydropathy profile, as well as the C-terminal region with positively charged amino acids. Our results supported that *atp8* may not be missing in the Mytilidae. Furthermore, we reconstructed the phylogenetic trees of Mytilidae and carried out positive selection analysis. The results showed that the deep-sea bathymodiolines experienced more relaxed evolutionary constraints. And signatures of positive selection were detected in *nad4* of *Limnoperna fortunei*, which may contribute to the survival and/or thriving of this species in freshwater.

## Supplementary Information

**Additional file 1:** **TableS1.** Branch-site model analyses in deep sea branches

## Data Availability

The datasets generated and/or analysed during the current study are available in the Genbank repository, accessions number: ON128252–ON128254.
