# Supplementary material for: Mitochondrial genomic analyses provide new insights into the “missing” atp8 and adaptive evolution of Mytilidae
Source: BMC Genomics. 2022 Nov 2;23:738. doi: 10.1186/s12864-022-08940-8 (PMC9628169; doi:10.1186/s12864-022-08940-8)
Supplement: Supplementary file 1 — Additional file 1: TableS1. Branch-site model analyses in deep sea branches [file 12864_2022_8940_MOESM1_ESM.docx]

Table S1 Branch-site model analyses in deep sea branches

| Genes | Model | lnL | 2△lnL | Parameter estimates | Positive sites | P-value |
| --- | --- | --- | --- | --- | --- | --- |
| *atp6* | Alternative | -9042.75 | 0 | *P*_0_ = 0.978 *P*_1_ = 0.008 *P*_2a_ = 0.014 *P*_2b_ = 0.000  ω_0_ = 0.012 ω_1_ = 1.000 ω_2_ = 1.000 | 49S(0.953) | 1 |
|  | Null | -9042.75 |  | *P*_0_ = 0.978 *P*_1_ = 0.008 *P*_2a_ = 0.014 *P*_2b_ = 0.000  ω_0_ = 0.012 ω_1_ = 1.000 ω_2_ = 1.000 |  |  |
| *cob* | Alternative | -21655.64 | 0 | *P*_0_ = 0.967 *P*_1_ = 0.009 *P*_2a_ = 0.023 *P*_2b_ = 0.000  ω_0_ = 0.018 ω_1_ = 1 ω_2_ = 1.000 | 321F(0.994) | 1 |
|  | Null | -21655.64 |  | *P*_0_ = 0.967 *P*_1_ = 0.009 *P*_2a_ = 0.023 *P*_2b_ = 0.000  ω_0_ = 0.018 ω_1_ = 1.000 ω_2_ = 1.000 |  |  |
| *nad2* | Alternative | -17777.81 | 0 | *P*_0_ = 0.961 *P*_1_ = 0.013 *P*_2a_ = 0.025 *P*_2b_ = 0.000  ω_0_ = 0.039 ω_1_ = 1.000 ω_2_ = 1.000 | 81S(0.982) 218M(1.000) | 1 |
|  | Null | -17777.81 |  | *P*_0_ = 0.961 *P*_1_ = 0.013 *P*_2a_ = 0.025 *P*_2b_ = 0.000  ω_0_ = 0.039 ω_1_ = 1.000 ω_2_ = 1.000 |  |  |
| *nad4* | Alternative | -21295.60 | 0 | *P*_0_ = 0.923 *P*_1_ = 0.046 *P*_2a_ = 0.030 *P*_2b_ = 0.001  ω_0_ = 0.036 ω_1_ = 1.000 ω_2_ = 1.000 | 267S(1.000) 268D(1.000) | 1 |
|  | Null | -21295.60 |  | *P*_0_ = 0.923 *P*_1_ = 0.046 *P*_2a_ = 0.030 *P*_2b_ = 0.001  ω_0_ = 0.036 ω_1_ = 1.000 ω_2_ = 1.000 |  |  |
| *nad5* | Alternative | -35059.64 | 0 | *P*_0_ = 0.887 *P*_1_ = 0.081 *P*_2a_ = 0.029 *P*_2b_ = 0.003  ω_0_ = 0.040 ω_1_ = 1.000 ω_2_ = 1.000 | 118V(0.999) 393M(1.000) | 1 |
|  | Null | -35059.64 |  | *P*_0_ = 0.887 *P*_1_ = 0.081 *P*_2a_ = 0.029 *P*_2b_ = 0.003  ω_0_ = 0.040 ω_1_ = 1.000 ω_2_ = 1.000 |  |  |
| *nad6* | Alternative | -6760.74 | 0 | *P*_0_ = 0.977 *P*_1_ = 0.011 *P*_2a_ = 0.011 *P*_2b_ = 0.000  ω_0_ = 0.022 ω_1_ = 1.000 ω_2_ = 1.000 | 23S(0.953) | 1 |
|  | Null | -6760.74 |  | *P*_0_ = 0.977 *P*_1_ = 0.011 *P*_2a_ = 0.011 *P*_2b_ = 0.000  ω_0_ = 0.022 ω_1_ = 1.000 ω_2_ = 1.000 |  |  |
